# Utilization of Expanded Criteria Donors in Liver Transplantation

**Published:** 2013-05-01

**Authors:** Reza F. Saidi

**Affiliations:** Division of Organ Transplantation, Department of Surgery, University of Massachusetts Medical School, Worcester, MA, USA

## Abstract

Improvements in surgical techniques, immunosuppression, and post-transplantation patient care have led to the optimization of liver transplantation outcomes. However, the waiting list for liver transplantation is increasing at a greater pace. The large gap between the growing pool of patients waiting for liver transplantation and the scarcity of donor organs has fueled efforts to maximize existing donors and identify new sources.

This article will be focused on the current state of liver transplantation using grafts from extended criteria donors (elderly donors, steatotic donors, donors with malignancies, donors with viral hepatitis) and from donation after cardiac death (DCD), as well as the use of partial grafts (split grafts and living-donor liver transplantation) and other suboptimal donors (donors with hypernatremia, infections, hypotension and inotropic support). Overall, broadened criteria for acceptable donor livers appear to lessen graft survival rates somewhat compared with rates for standard criteria organs.

## INTRODUCTION

Liver transplantation (LT) is the treatment of choice for patients with end-stage liver disease. Improvements in surgical techniques, immunosuppression, and patient management have led to the optimization of liver transplantation outcomes. A major challenge for the transplant community is to develop strategies to close the gap between the number of patients in need of a transplant and the number of available organs. LT is unquestionably the preferred therapy for most patients with end-stage liver failure since both survival and quality of life are superior in allograft recipients compared to similar patients without LT [1]. As outcomes of transplantation have improved, the number of transplant candidates listed for deceased donor (DD) transplantation has increased dramatically over the years. One of the main strategies to address the discrepancy between supply and demand in organ transplantation is expansion of the DD kidney pool utilizing expanded criteria donor (ECD) and donation after cardiac death (DCD) donors [1-3]. This has been a major focus of the US Department of Health and Human Services organ donation breakthrough collaborative which was initiated in 2003, with the objective of increasing access to transplantable organs. Scientists, clinical and organ procurement agencies are expanding the donor pool through two mechanisms. The first mechanism is using organs that were previously thought to be associated with a high risk of primary nonfunction (PNF) or delayed graft function (DGF), the so-called ECD or marginal livers [3-4] (*i.e.*, donors with steatosis, with malignancies, with viral infections, older or elderly donors, DCD, *etc*). These livers considered unacceptable for transplantation in the past, are now being transplanted, but the main difficulty is in defining the criteria. The second way to expand the donor pool is through advances in medical practice, particulary surgical techniques including split liver transplantation (SLT) and living donor liver transplantation (LDLT). Although the ECD organs may not be optimal, the high death rate on the waiting lists produced a stark choice between dying without a liver or proceeding with a liver that was perhaps not ideal [1-4]. It is known that the marginal grafts exhibit poor tolerance to ischemia/reperfusion (I/R) injury, which is an important cause of liver damage occurring during surgical procedures including hepatic resections and LT [3]. Also, I/R injury is the underlying cause of graft dysfunction in ECD organs [1]. Moreover, I/R affects negatively the process of liver regeneration in surgical conditions including hepatic resections and small-for-size LT [3].

**ECD LIVERS FOR TRANSPLANTATION**

ECD liver could be defined as an organ with an increased risk of PNF or DGF that may cause higher risks of morbidity or mortality in the recipient. However, there is no consensus about the specific factors that define a graft as ECD or about which factors or combinations thereof should exclude the graft from being used because of unacceptable risk to the recipient [3, 4]. Some of the ECD liver used are obesity (weight >100 kg or BMI >27 kg/m^2^); age >50 years; macrovesicular steatosis >50%; intensive care unit stay >4 days; cold ischemia time >14 h; prolonged hypotensive episodes of >1 h, and <60 mm Hg with high inotropic support (dopamine >14 μg/kg/min); hypernatremia (peak serum sodium >155 mEq/L); viral infections; sepsis; alcoholism; extrahepatic neoplasia; gender mismatch or DCD [3-7]. 

The severity of the resulting liver dysfunction is also determined in part by the degree of hepatic injury that occurs as a consequence of local and systemic hemodynamic changes in response to brain death, liver retrieval and implantation. These factors crucially influence the graft viability [6].

Broadly, there are two categories of ECD livers [4]. Firstly, there are livers that carry a high risk of technical complications and impaired function (*i.e.*, steatotic donors, DCD, elderly donors, split livers, and donors with high inotropic requirement). Secondly, grafts will be considered marginal, if they carry a risk of transmission of infection or malignancy to the recipient (*i.e.*, donor with viral infections or donors with malignancy) (Table 1).

**Table 1:** Different types of ECD liver

**ECD Allograft **
Steatotic donors
Elderly or older donors
Non-heart beating donors
Donors with hypernatremia
Donors with hypotension
Donors with viral infections
Donors with malignancies
Donors with infections
**Technical Variant Graft **
Living-donor liver transplantation

Despite numerous retrospective studies, the impact of each donor variable on graft function and recipient survival is still under investigation because of the contradictory results. Some investigators have indicated comparable results regarding graft function and patient survival after transplantation of marginal donors *vs*. standard grafts, but most reports support a clear correlation between graft quality and post-transplantation outcome. New concepts, especially the extended criteria donors scoring system developed by Cameron, *et al*, [7] and the donor risk index (DRI) proposed by Feng, *et al*, [8] have allowed a more integral and quantitative assessment of the impact of extended donor criteria on post-transplantation mortality and the risk of graft failure. According to Cameron, *et al*, [7] a donor older than 55 years, donor hospital stay more than five days, cold ischemia time more than 10 h, and warm ischemia time more than 40 minutes contribute significantly to recipient mortality and were assigned one score point each. 

Feng, *et al*, [8] analyzed 20,000 transplants from the Scientific Registry of Transplant Recipients (SRTR) database and developed a DRI, which is calculated from seven donor and two transplant variables that were found to be independently associated with an increased risk of graft failure. These included donor >40 years, donor height, donation after cardiac death, split/partial grafts, cerebrovascular accident or other cause of death (except trauma, stroke, or anoxia), cold ischemia time, and organ sharing outside the local donor service area. Although a conclusive statement on the impact of graft steatosis could not be made due to incomplete data in the registry, the analysis of Feng, *et al*, [8] highlights the relevant donor risk factors and supports a clear correlation between organ quality and post-transplantation outcome. 

Actually, the use and acceptance of ECD livers vary among various transplant centers [9]; therefore, the decision to transplant a specific organ depends on the judgment of the transplant surgeon and consideration of the specific recipients, but even the consequence of using ECD allografts in a future remains unclear.

**TYPES OF ECD LIVERS**

**Steatotic donors**

Hepatic steatosis is frequent in deceased organ retrievals and live donors, and reported in 9%–26% of donors [10-12]. Given the steady increase in the mean age of DD and the overall increase in the prevalence of obesity, it is expected a further increase in the prevalence of steatosis in both DD and living donors [13]. This represents a large potential pool of donors. The potential use of steatotic livers for transplant has become a major focus of investigation. However, the clinical problem is still unresolved since steatotic livers are more susceptible to I/R injury and, when used, have poorer outcome than nonsteatotic livers. Indeed, the use of steatotic liver for transplantation has been associated with increased incidence of PNF [5, 10, 14] and DGF [15]. Moreover, nearly one-third of all donated livers are discarded because their pathological conditions, hence accentuating the problem in the shortage of organs [2, 16]. Therefore, minimizing the adverse effects of I/R injury could improve outcomes in steatotic liver surgery, increase the number of both suitable transplantation grafts and patients who successfully recover from LT.

Some early studies showed that graft steatosis is the most important variable in multivariate analysis of factors determining graft function after transplantation [17]. However, steatotic livers can be transplanted safely with good results for long-term organ survival, especially if other contraindications for their use are absent [5, 10, 14].

The causes of hepatic steatosis are variable and include obesity, older age, alcoholism, diabetes mellitus, hyperlipidemia and postmortem nutritional changes [11]. Histological patterns showed that there are two forms of steatosis encountered in liver grafts: 1) macrovesicular steatosis in which the fat vacuoles occupy most of the hepatocytes cytoplasm and displace the nucleus peripherally, and considered a more dangerous lesion; and 2) microvesicular steatosis, where the vacuoles are smaller and have a centrilobular distribution, which is commonly found in pathological conditions associated with mitochondrial injury such as some metabolic disorders; it is largely reversible and does not tend to cause harmful post-transplantation consequences [18]. Severity of steatosis is traditionally graded as mild <30%, moderate 30%–60%, and severe >60%. It has been shown that a scoring system that includes degree of steatosis and donor age correlates well with the outcome of fatty livers [19]. 

The transplantation outcomes are not affected by hepatic microsteatosis, regardless of its severity, and adequate function of livers has been reported [20]. In addition, liver allografts with mild macrosteatosis (<30%) can be safely used, assuming there are no other donor or recipient risk factors, because these livers show similar results to nonsteatotic grafts [21]. Liver allografts with severe macrosteatosis (>60%) have a significant risk of graft failure and should not be used for transplantation, unless there is an urgent situation when they are used as a bridge [11]. The use of grafts with moderate steatosis (30%–60%) is controversial, because these may impose a relative risk on post-transplantation outcomes. Previous reports have shown an increased incidence of PNF after LT from donors with moderate steatosis compared with nonsteatotic livers (13% *vs*. 3%) [22]. 

In the transplant setting, a method for determining the extent of steatosis remains imprecise and inconsistently reported. In particular, the distinction between macrovesicular and microvesicular steatosis is often cited as important, but the precise definition of these, their macroscopic and microscopic assessment, and their relative quantification depend on the histological technique and the experience of the interpreting pathologist [23]. On gross examination of the liver, fatty livers are often yellow and contain blunted or round edges, in contrast to the more normal salmon color and sharp borders. However, for the moment, microscopic assessment of the liver remains the “gold standard” for the diagnosis and quantitation of steatosis [4]. Liver biopsy of the donor liver and frozen section is the preferred method because of time constraints between graft retrieval and transplantation [24] and is considered to be mandatory in certain settings. 

Recent studies have shown that ultrasonography, CT and MRI all display a reasonably good specificity for the diagnosis of steatosis; they also have an unacceptably low sensitivity compared to histology, with the only exception for those with massive steatosis. Unfortunately, the current imaging modalities are inaccurate and inadequate in the quantitation of liver steatosis and do not distinguish clearly between the microvesicular and the macrovesicular types [25]. Other tools like biomechanical impedance and transient elastography (fibroscan) have been shown to predict steatosis/fibrosis, and their use may be extended for the assessment of the donor liver [26]. With increasing the prevalence of steatosis in the donor population, more surrogate markers of organ quality are needed. 

**Elderly or older donors**

Donor age steadily increased over recent decades. In 1994, only 20% of deceased donors were 50 years or older. This percentage increased by more than 150% in the year 2004 [27]. Initially, donor age >50 years was considered a contraindication to liver donation because it was thought to be associated with poor graft outcomes, although some studies suggested that donors older than 50 years without additional risk factors have similar outcomes compared to younger donors [28, 29]. Therefore, given these later results, age, itself, should not be a contraindication to liver donation. However, a recent study [30] reported that liver grafts from donors >70 years of age had a relative risk of 1.4 and 1.7 for long-term graft failure and mortality, respectively. More recent studies using the large databases of either SRTR/United Network for Organ Sharing (UNOS) or European Liver Transplantation Registry (ELTR) clearly identified donor age as an important risk factor for poor outcome after LT [31]. 

In contrast to other organs, the liver may be more immune to senescence, particularly in an otherwise healthy person. This is possibly because of the liver’s large functional reserve, regenerative capacity, and dual blood supply, which exceed its metabolic needs [32, 33]. On the other hand, older donor livers tend to be smaller (in weight and volumen) and darker-colored, and may have developed fibrous thickening of the capsule [34] than younger livers; also, blood flow is reduced with aging [35]. Whether these morphological changes impact on organ function after transplantation remains to be elucidated. It has been shown that older donor livers are more susceptible to endothelial cell injury from cold ischemia and show decreased ATP synthesis after reperfusion, which may influence the decreased regenerative capacity [36] and synthetic function [37].

Some factors including steatosis or prolonged ischemia could contribute to the poor post-transplantation outcomes from elderly donors [38]. Attention should be paid to the possible effects of atherosclerosis on arteries. Calcified plaques on the hepatic artery might result in severe complications [39]. Elderly donor also appears to have an additive adverse effect on liver recipients with hepatitis C virus (HCV). [40]. Also, donor age may be important in recipients with primary biliary cirrhosis as this can adversely affect their outcome [41]. Transmission of malignancy is another consideration with aged donors because of the higher incidence of unrecognized malignancies in the elderly [42].

**Donation after cardiac death**

Although live donors and donation after brain death (DBD) account for the majority of organ donors, in the recent years, there has been a growing interest in donors who have severe and irreversible brain injuries but do not meet the criteria for brain death. If the physician and family agree that the patient has no chance of recovery to a meaningful life, life support can be discontinued and the patient can be allowed to progress to circulatory arrest and then still donate organs—donation after cardiac death (DCD). In the last 10 years, the number of deceased organ donors nationally has increased modestly, whereas DCD has increased 10-fold with over 900 cases of DCD reported in 2009 [2]. 

Consistent with the goals set by Health Resources and Services Administration (HRSA) for DCD development, the percentage of donors from DCD continues to increase. There has been a significant increase in the percentage of donors that are categorized as DCD from 8% in 2006 to 9.8% in 2007; the number and percentages of DCD liver and kidney transplants continue to increase substantially [2]. In a recent study, we examined the pattern of donation and utilization in the United States using Organ Procurement and Transplantation Network (OPTN)/UNOS database of individuals who were consented for and progressed to organ donation between January 2001 and December 2010. We encountered parallel changes in this study with increasing the number of DCD donors from 3.5% in era-1 (2001–2005) to 9.3% in era-2 (2006–2010) [2]. On the other hand, we noted a decrease in living donation. Although the total number of deceased donors did increase by 25% from era-1 to era-2, the number of DBD donors that peaked in 2006, constantly decreased since. The main reason for the increase in the number of deceased donors was the rapid expansion of the DCD group which rose 230% when comparing era-1 (n=1135) with era-2 (n=3748). At the same time, the number of DBD donors increased by only 17% when comparing era-1 and era-2 [2]. Whether this represents addition of donors who would not have ever progressed to brain death or an exchange for DCD in cases that would have previously followed a DBD pathway still remains uncertain. In case of the latter, this may indicate the occurrence of a change in clinical practice in which withdrawal of support is offered earlier in the patient’s course, before brain death has occurred. Saidi, *et al*, [43] identified a significant change in resuscitative practices over time, with a striking rise in new surgical interventions such as craniostomy, craniotomy, cooling, *etc*, that have the potential to intercede in the progression to brain death. These interventions were strongly associated with intent to donate via DCD. The lesser likelihood of making the diagnosis of brain death in these patients provides a plausible explanation for at least part of the stagnant growth of DBD compared with DCD in the national data. 

As a result of increasing utilization of DCD donors, more donors with comorbities and elderly donors, we also noted a dramatic increase in the discard rates. Overall, discard rate increased from 13,411 (11.5%) in era-1 to 19,516 (13.7%) in era-2. This increase in discards was especially prominent in the DCD group which rose from 440 (20.9%) in era-1 to 2,089 (24.9%) in era-2 [2]. The discard rate for DCD livers has increased. We noted 78% increase in the discard rate of DCD livers, although, the discard rates for DBD livers and kidneys remained stable [2].

Inspection of UNOS data reveals that nationally in 2009, an average of 3.6 organs were recovered from DBD donors compared to 2.5 organs from DCD; the consented DCD donors who did not progress were not considered. In addition, 3.1 organs were transplanted form DBD donors compared to 1.9 from DCD. On average per 100 donors, DCD donate 20 less kidney (170 *vs*. 190), 40 less liver (40 *vs*. 80), and five less pancreas (2 *vs*. 7) when compare to DBD [2].

It should also be clearly noted that our intent is not to challenge the standard of care or ICU management of patients with severe head injury; rather we have attempted to clarify and recognize the impact these new therapies and practice shifts have on the opportunity for organ recovery from deceased donors. We do recognize however, that there may be specific cases in which there exists a choice to withdraw mechanical, ventilated or organ-perfusion support immediately or to determine if the potential donor will progress to brain death in a timely fashion. In such cases, if the dying patient had expressed a premortem intent to be an organ donor to help others, and if the ability to make the diagnosis of brain death may be imminent, we suggest that it may be appropriate to include in the end-of-life discussion with the next-of-kin the implications of withdrawal of mechanical, ventilated or organ-perfusion support on the nature and magnitude of the gift that was intended by their loved one. We fully understand that organ donation might not be the first or foremost issue on the mind of family or intensive care physicians. However, after the decision to offer withdrawal of mechanical, ventilated or organ-perfusion support has been made and the discussion held with the next-of-kin, we contend that the potential donor's end-of-life wishes regarding organ donation should be given due consideration. In the appropriate circumstances, the impact of DCD *vs*. potential DBD pathways on the magnitude and nature of the resulting gift might be a reasonable component of the end-of-life discussion. Some families have decided to discontinue mechanical, ventilated or organ-perfusion support as soon as possible but others encouragingly have agreed to wait to fully honor the wishes of the dying potential donor, to maximize the opportunity of organ transplantation after brain death.

The data on marginal organs are compounded by the large and ever-growing concerns about post-transplantation outcomes. Allograft and patient survival of DCD kidneys are reported to be similar to DBD kidneys, but DCD kidneys have been associated with increased resource utilization [44]. Saidi, *et al*, [44] showed that ECD and DCD kidneys are associated with more frequent need for hemodialysis after transplantation, longer length of stay, more hospital re-admissions due to poor or late-onset graft function and more CMV infections in recipients of ECD and DCD kidneys which resulted in a US$ 20,000–25,000 higher cost for their initial medical care and economic pressure on the transplant centers [44]. For DCD livers, there is a high rate of biliary strictures that have been attributed to the period of warm ischemia that occurs between withdrawal of donor life support and organ preservation. This leads to a reduction in graft survival and an increase in the need for retransplantation [45]. On the other hand, marginal liver allografts has been shown to be associated with increased hospital costs [45]. The concern about overall outcomes and cost of utilization of marginal organs can impact the decision of physicians and transplant center to use these organs. The transplant community must also monitor the effects of changes in organ procurement practices, especially defining optimal identification and management of marginal donors and more investment in live donation. There should also be an emphasize on measures to improve the quality of marginal organs such as *ex vivo* preservation methods or extracorporeal support for donors after cardiac death to assess viability and provide resuscitation of DCD and ECD organs. Organ allocation and distribution has its roots in the heterogeneous and somewhat arbitrary geographic boundaries that determine the current donation service areas (DSA) and UNOS regions. This has led some to call for broader allocation units to make distribution more equitable and not based so tightly on geography. This can potentially lead to better utilization of organs and also decrease the discard rate. It has been showed that there was a wide variation in different regions regarding changes in organ recovery, transplantation and discards [46-49].

DCD are divided into “controlled” and “uncontrolled” donation based on Maastricht classification in order to underline differences in clinical practice and graft outcome. Controlled donations occurs with a circulatory arrest after planned withdrawal of life support equipment, most often in an ICU in a controlled environment with a donor surgical team available. In uncontrolled donation, the donor death occurs completely unplanned, outside the hospital or in an emergency room following an unplanned cardiac arrest with unsuccessful attempt of resuscitation [50, 51]. In controlled DCD, warm ischemia time can be accurately assessed, and cold ischemia can be minimized; therefore, the organs are comparatively far less prone to ischemic damage and tend to offer superior post-transplantation function [51]. This was not the case for uncontrolled DCD since in this clinical situation the organs suffer severe ischemic insult. Liver allograft survival from uncontrolled DCD is poor (17%–41%) [52]. 

Ischemic time has been shown to be extremely important when DCD is considered [53]. If warm ischemic time is restricted to <30 minutes and cold ischemia time <10 h, graft survival rate in the DCD group was found to be 81% and 67% at 1 and 3 years, respectively, which is not significantly different from recipients of dead-brain donors [54]. Results from uncontrolled DCD were less good, being graft survival at 2 years of 55%. The use of uncontrolled DCD livers was also associated with significantly higher incidence of PNF, DGF and biliary complications [55-59]. 

It is likely that further refinements in patient selection, operative technique and preservation solutions will improve the results and utility of non-heart-beating donation (NHBD) and potentially expand the donor pool by 20%–30% [59].

**Donors with hypernatremia**

Hypernatremia was shown to be one of five variables with prognostic value in predicting graft survival after transplantation [60]. Some studies have suggested that donors with hypernatremia can affect graft function and increase the risk of graft loss [61]. The mechanism for the deleterious effect of elevated donor sodium on graft function is thought to be a result of cell swelling, increased osmolality and exacerbation of reperfusion-mediated injury [62]. The cause of hypernatremia could be related to derangement of fluid balance and diabetes insipidus in potential donors [61]. In a study investigating the peak donor sodium level and the corrected sodium level at the time of retrieval, it was found that hypernatremia (sodium >155 mEq/L) was associated with 18.5% rate of PNF compared with 3.4% in eunatremic group. With the correction of hypernatremia before procurement, this rise in the PNF was no longer found [62]. Another pilot study at University of California examined the effects of infusing 5% dextrose (D_5_W) in water through the inferior mesenteric vein before harvesting the organ if the donor sodium level was >160 mEq/L. In the 17 donors who received D_5_W to decrease hypernatremia, the rates of DGF/PNF were 0% compared with a group of historical controls that experienced a 60% incident of PNF/DGF [62].

**Donors with hypotension and inotropic support**

Previous UNOS data have shown that donor organs subjected to prolonged hypotension have no significant increase in post-transplantation graft loss. However, graft loss was increased in liver transplant recipients when donors received norepinephrine [61]. In other studies, dopamine dose >10 μg/kg/min [50] or 6 μg/kg/min [62] had a significant effect on early graft function. However, other factors such as age and fat content may modify these effects in either direction.

Briceño, *et al*, [61] reported that unstable donors with high doses of inotropic drugs have an increase in severe preservation damage rate, and trends to normalize hemodynamic status in dead-brain donors did not correct liver dysfunction. Probably, time-dependent administrations of high-dose dopamine and epinephrine have a harmful effect on liver function.

**Donors with viral infections**

Potential donors with positive viral infections should not be completely ruled out from the donor pool [63]. Viral infections such as hepatitis B, and hepatitis C are routinely screened in potential donors and are frequently knowingly transmitted because, for the most part, there are effective treatments for these viruses in immunosuppressed hosts. Thus, despite a relatively efficient transmission of these viruses and documented deaths that are directly related to them, donors testing positive for these viruses are routinely considered suitable [64].

<subsub>Hepatitis B virus (HBV)

Approximately 5% of people worldwide are chronically infected with hepatitis B. Overall, 15% of those chronically infected go on to develop cirrhosis, and an additional 20% will require LT. Acquisition of the HBV remains a concern after LT because the majority of the infections occur via transmission by the donor liver [65], but some donors with past exposure to HBV infection can be used selectively in some recipients.

Donors who are hepatitis B surface antigen negative (HBsAg^–^) but hepatitis B core antigen positive (anti-HBc^+^) have transmitted HBV infection to liver recipients who are HBsAg^–^ at a rate of 33% to 78% [66]. Early studies of the use of hepatitis B core antibody positive allografts to treat HBV^+^ recipients suggested that the risk of HBV transmission was extremely high and carried a high mortality. However, in patients who are immune to HBV (previous vaccination) it has been found to be safe to use these organs [67]. In recipients with active HBV infection or in desperate circumstances, these organs have been used safely in combination with antiviral prophylaxis and immunnoglobulins [64-68]. Additionally, donors with positive hepatitis B surface antibody (anti-HBs) do not appear to transmit HBV infection after LT [69].

The development of combined prophylaxis with hepatitis B immune globulin (HBIg) and lamivudine has proved effective not only against HBV recurrence but also against *de-novo* HBV infection or transmission in recipients of anti-HBcAb^+^ livers [67-69]. Nery, *et al*, [68] reported that of 62 recipients of anti-HBc^+^ livers, 60 were serologically free of HBV infection under combined or lamivudine monotherapy. These data suggest that the use of HBcAb^+^ grafts is comparable with core antibody negative grafts and that survival was improved with dual immunoprophylaxis [64-68]. In addition, Prieto, *et al*, [63] reported that post-transplantation HBV infection developed in 15 of 30 recipients of livers from anti-HBcAb^+^ donors compared with 3 of 181 livers from anti-HBcAb donors. Recipients of livers from anti-HBc^+^ donors are at high risk for acquiring HBV infection, whereas recipients of livers from anti-HBs^+^ donors are significantly less likely to acquire HBV infection, and this latter group may play a role in expanding the donor pool [63].

Another possibility to increase the organ pool is to use grafts from HBsAg^+^ donors. There are several studies which suggested that LT from HBsAg^+^ donors seems to be a safe procedure in the era of highly effective antiviral therapy [70, 71].

<subsub>Hepatitis C virus (HCV)

About 5% of all potential organ donors are positive for antibody to HCV [72], and the transplantation because of HCV cirrhosis has increased because of the greater prevalence of the virus in the last 15 years [73]. Initially, the use of HCV^+^ donor organs in LT was a source of great controversy and not commonly practiced. Underlying this practice was a concern for increased risk of aggressive viral recurrence in patients receiving HCV^+^ grafts. LT for recipients with HCV cirrhosis from HCV^+^ donors were found to provide graft survival that is equivalent to HCV^–^ grafts to HCV^+^ recipients [74]. Short-term studies in the early 1990s showed no difference in outcomes of HCV^+^ grafts; increasing donor shortage allowed for the use of HCV^+^ donor grafts in recipients with HCV to expand the donor pool. Long-term follow-up in the late 1990s confirmed that the use of grafts from HCV^+^ donors is safe and that patient and graft survival are not affected [75]. Recurrence rates of hepatitis C, manifested by mild chronic hepatitis, fibrosis, or cirrhosis have been reported to be 54.55% in HCV^+^ donor grafts when compared with 41.74% in HCV^–^ grafts. Patient and graft survival at 4 years post-transplantation in HCV^+^ donor grafts have been shown to be 83.9% and 71.9% *vs*. 79.1% and 76.2%, respectively, in HCV^–^ donor grafts [76]. Similar rates of HCV recurrence, patient survival, and graft survival have been reported by different centers using HCV^+^ liver grafts for patients requiring transplantation for HCV cirrhosis [74]. Moreover, in an report by Marroquin, *et al*, [75] showed that patient survival at two years was significantly higher in HCV^+^ recipients of HCV^+^ grafts than in HCV^+^ recipients of HCV^−^ grafts (90% *vs*. 77%). In contrast, other studies indicated that in patients with HCV-related liver disease, there was no significant patient survival difference between the patients who received HCV^+^ grafts and who received HCV^−^ grafts [76].

In general, it is obvious that livers from donors with active ongoing hepatitis and/or fibrosis should not be used for transplantation. In donors with a history of such infection, there have been recommendations for a routine liver biopsy before use of a graft for transplantation. A scoring system has been proposed to aid making the decision of whether a graft should be used for transplantation in this setting [77].

**Donor with malignancies**

The transmission of donor-derived malignancies to recipients with catastrophic outcomes has been reported [78]. Certain tumor types such as glioblastoma, astrocytomas and medulloblastoma, as well as tumors that have breached the blood-brain barrier following ventriculoperitoneal shunts or surgery along with cerebellar tumors and previous prolonged chemotherapy for such tumors carry a higher risk of transmission and should be avoided unless the recipient status warrants the extra-risk [79, 80]. 

According to UNOS database, 2.7% of deceased donors have a history of cancer. Between 2000 and 2005, grafts from donors with a history of malignancy were used in 891 liver transplantations. The most common cancers were nonmelanoma skin cancer (n=306) followed by the central nervous system (CNS) malignancies (n=179) and carcinoma of the uterine cervix (n=108). Forty-five donors had a history of melanoma [73]. Presumably, none of the donors had any evidence of active malignancy, with the exception of nonmelanoma skin cancers such as basal cell carcinoma and squamous cell carcinoma and the CNS malignancy. During the study period, only two donors transmitted a fatal malignancy to recipients [81]. Given earlier reports, livers from donors with a history of melanoma or gliobastoma should not be used for transplantation [81, 82].

Buell, *et al*, [83] have reported an overall transmission rate of the CNS tumors of 23%. If donors have high-grade malignancies and/or risk factors, recipients face an increased incidence of tumor transmission of 53%. Risk factors include surgical shunts, previous craniotomy, or previous prolonged chemotherapy. These high-risk donors also should be avoided. It is left to the judgement of the transplanting team that determine the use of these organs under certain circumstances.

**TECHNICAL VARIANT GRAFTS**

In an attempt to expand the size of the donor pool, a number of surgical techniques have been developed over the past 15 years, including SLT and LDLT [84]. Couinaud’s [85] anatomical classification that later refined by Bismuth [86], permits the creation of partial liver grafts from either deceased or living donors. Couinaud’s classification divides the liver into eight independent segments, each of which has its own vascular inflow, outflow, and biliary drainage [85]. Segments IV to VIII are used for adults, whereas left lateral lobes (segments II and III) or left lobes (segments II, III, and IV) are used for pediatric recipients. Bleeding, bilomas, and portal vein thrombosis are complications related to the procedure itself, which are associated with an increased number of re-operation.

**Living-donor liver transplantation**

Living-donor liver transplantation (LDLT) is an established treatment for end-stage liver disease. In Asian countries; approximately 90% of donor organs for liver transplantation are from live donors, as deceased donor rate is low due to social and religious factors. The peak of adult LDLT was in 2001, but the sudden death of a living donor postoperatively in New York led to constant decrease in the number of LDLT in the United States. The decline in live donors could be due to loss of income while off work after the procedure, potential future insurability issues, and expenses that may not be covered by insurance or risk of donor’s complication/death [87]. 

LDLT has some well-documented advantages including the use of a graft from a healthy donor with minimal ischemic time, the ability to schedule surgery electively, a reduced risk of the recipient dying on the waiting list, and allowing the recipient to be medically stabilized. LDLT has disadvantages as well: a higher rate of surgical complications for both the donor and recipient, and a potential risk of small-for-size syndrome. Furthermore, LDLT carries inherent risks for the healthy donor. Therefore, careful selection of the donor and recipient is crucial to minimize risks and complications and to obtain acceptable outcomes [88-91].

Initially, donors undergo psychosocial evaluation to assure no coercion. Donors are then evaluated by clinical examination and serologic testing for liver disease, renal disease, viral hepatitis, and HIV. The second stage is comprised of diagnostic studies to evaluate the vascular and biliary anatomy of the donor. Several options for preoperative imaging are available. These include non-invasive modalities such as multi-phase CT, duplex ultrasonography, and MRI. The third phase can consist of percutaneous liver biopsy. Many centers will perform liver biopsy routinely or selectively.

The ideal candidates for LDLT are usually those patients who are not extremely sick from end-stage liver disease typically with MELD scores <20. One of the most difficult problems to tackle in the expansion of LDLT to adults is graft size to avoid small-for-size syndrome. This is manifested as the constellation of persistent ascites, coagulopathy, prolonged cholestasis, and poor bile production, in the absence of a technical cause. 

The pathophysiology of small-for-size syndrome is not well described but the main cause seems to be related to allograft size, portal hyperperfusion or venous outflow obstruction. The graft to recipient weight ratio (GRWR) should be at least 0.8% [88-90].

The Adult-to-Adult Living Donor Liver Transplantation Cohort Study (A2ALL) is a consortium of the United States liver transplant centers with the primary goal of comparing outcomes of adult-to-adult LDLT *vs*. DDLT. In its first detailed report on 385 cases, 90-day and 1-year graft survivals were 87% and 81%, respectively. The outcomes were characterized by frequent biliary complications (30% early, 11% late) and 13% graft failure because of vascular complication, PNF, and sepsis. Marcos, *et al*, compared the outcomes after adult-to-adult LDLT to those after DDLT using nationwide databases. The 1- and 3-year patient survival rates after LDLT were similar to those after DDLT (89.1% and 80.3% *vs*. 85.7% and 77.7%, respectively). Graft survival rates at one and three years were also similar (79.3% and 70.1% *vs*. 80.7% and 71.1%, respectively). However, the severity of illness was substantially lower in LDLT recipients than in DDLT recipients [88].

It has been suggested that HCV replication might be increased in reduced-size LDLT grafts, but the data is controversial. The major concern in adult-to-adult LDLT is the adequacy of graft size. Although harvesting a larger graft carries a higher risk for the donor, a residual liver volume of 30% can be tolerated by the donor in the absence of steatosis and right lobe grafts have become standard for adult LDLT [89-91].

To minimize the donor risk, using left lobe has been popularize in the United States and Asia. Although, single center data showed comparable outcome using right lobe *vs*. left lower, analyzing the United States experience revealed that lower allograft and patients survival using left lobes due to high rate of complication and need for retransplantaion [91].

**Split-liver transplantation**

In split-liver transplantation (SLT) two allografts are created from a single deceased donor liver allograft. This technique was developed to address the organ shortage. However, the technical and logistic issues in both donors and recipients prevent its worldwide usage. SLT accounts for only 4% of liver transplantations in the United States. While splitting was originally performed as an *ex vivo* bench procedure, *in situ* liver splitting was introduced to decrease CIT and prevent blood loss after reperfusion. It had been feared that prolonged surgical time and increased blood loss associated with *in situ* splitting of livers might negatively affect the function of other solid organs procured from the same donor, but in fact, in stable donors, *in situ* splitting can be accomplished without significant negative effects on other organs.

Left lateral segment (LLS) or left split grafts have mainly been transplanted into children, and right split or right trisegment (RTS) grafts into adults, with excellent outcomes. Rogiers, *et al*, [92] reported on 100 livers that were split *in situ*, yielding 190 grafts for transplantation. LLS grafts were transplanted to pediatric recipients and RTS grafts were transplanted to older children and adults. Patient and graft survivals were equal to those in 1086 recipients who received whole liver from deceased donors [92, 93].
